# A Layman's Letter Answered

**Published:** 1921-07-16

**Authors:** F. Alex


### "The Letters of a Layman."

*To the Editor of* The Hospital.

### A Layman's Letter Answered.

*To the Editor of* The Hospital.

Sir,—To quote "Ierne," the man who knows least about his patients is the man of medicine. If such a statement could be seriously taken as representative of any considerable body of lay opinion, it might be well also to advise the compulsory obliteration of the greater part of the medical profession and most of its institutions. Evidently "Ierne" would not go so far. But the man of medicine and his ways incense him, he sees but the fringe of the great profession he would criticise, and therefore without hesitation, and unconscious of his fallacies, plunges to the attack.

One who has trodden a few at least of the diverse paths of medical endeavour may be excused perhaps for dissecting some of the headlong statements in this "Letter of a Layman." First, what precisely does "Ierne" mean by a "man of medicine"? As one reads his comments the inevitable conclusion is that one type only is considered—namely, the general practitioner. It is true that this type contains the majority, much of the youth, and indeed many of the best of the profession. Their defence can come later, but let the public at all costs remember the consultant surgeons, physicians, pathologists, public health and institutional officers, and the vast cosmos of professional elements welded under the Voluntary Hospital principle. Have these professional elements done nothing to stimulate the layman's education in health matters? Surely "Ierne" cannot have missed the enormous amount of health propaganda to which the country has been officially and unofficially subjected during the past two years. Even individual hospitals and staffs have gone to great expense and trouble, practically and publicly, to demonstrate the principles of scientific hygiene. Even at the Efficiency Exhibition they were there in force. But "Ierne" has missed all these elements, and the work achieved. Their justification need not detain us. It is daily self-evident.

Instead, he concentrates on the private general practitioner. One states "private" because, if "Ierne's" "man of medicine" is a panel practitioner, there is no indication of the fact in his argument, and his criticism has apparently nothing to do with the weakness of the National Health Insurance schemes. In vague words one reads that the doctor "tries his best, no doubt, to cure a patient when he is sick. But he makes no effort, nor does he consider it part of his duty to keep him in good health when he is well." To this, if it is intended as a generalisation, there is but one reply—a flat contradiction. No modern medical man worthy of the name is oblivious, in theory or in practice, of the common elements of preventive medicine. Since, and indeed long before, the Armistice, professional meetings and journals have been replete with discussions and cultivations of the Preventive Principle. The Beginnings of Disease has become a veritable catchword in the Press. Look where we will we find medical education, the new hospital-teaching units, institutes like that of St. Andrews, all tending towards one great educative goal—the Prevention of Disease. And yet, in spite of all this, "Ierne," a layman, now "discovers" that what the doctor ought to do is to prevent his patient from becoming ill. The fact that "Ierne" *has* discovered it is more indicative of progress in that very phase of public education which he urges the doctor to carry out, than proof that it does not exist. For years the doctor has been urging the layman to appreciate the value of prevention, and periodical medical examination, the danger of neglect, and the urgent call for improved national hygiene. As a doctor, one would applaud "Ierne's" discovery, if he did not suggest that the man of medicine neglects to prevent illness because such action "would not butter the doctor's parsnips." If the whole criticism were not absurd, it might, as here, be hurtful. Fortunately and cautiously, "Ierne" concludes, "So far as I am aware there is at present no means of convincing the public of the importance of constant watchfulness." Obviously he is not aware of them, but he can rest assured that they exist. Does he read The Hospital's own weekly review of these public matters, as well as write the "Letters of a Layman"?

Yours truly,

F. Alex.

